# Special Packaging Materials from Recycled PET and Metallic Nano-Powders

**DOI:** 10.3390/polym15153161

**Published:** 2023-07-25

**Authors:** Romeo C. Ciobanu, Mihaela Aradoaei, Alina R. Caramitu, Ioana Ion, Cristina M. Schreiner, Violeta Tsakiris, Virgil Marinescu, Elena Gabriela Hitruc, Magdalena Aflori

**Affiliations:** 1 Department of Electrical Measurements and Materials, Gheorghe Asachi Technical University, 700050 Iasi, Romania; mihaela.aradoaei@academic.tuiasi.ro (M.A.); cristina-mihaela.schreiner@academic.tuiasi.ro (C.M.S.); 2 National Institute for Research and Development in Electrical Engineering ICPE—CA, 030138 Bucharest, Romania; alina.caramitu@icpe-ca.ro (A.R.C.); ioana.ion@icpe-ca.ro (I.I.); violeta.tsakiris@icpe-ca.ro (V.T.); virgil.marinescu@icpe-ca.ro (V.M.); 3 “Petru Poni” Institute of Macromolecular Chemistry, 41A Gr. Ghica Voda Alley, 700487 Iasi, Romania; gabihit@icmpp.ro

**Keywords:** polyethylene terephthalate, polypropylene, Al nanopowder, Fe nanopowder, drugs packaging

## Abstract

The European methodology for plastics, as a feature of the EU’s circular economy activity plan, ought to support the decrease in plastic waste. The improvement of recycled plastics’ economics and quality is one important part of this action plan. Additionally, achieving the requirement that all plastic packaging sold in the EU by 2030 be recyclable or reusable is an important objective. This means that food packaging materials should be recycled in a closed loop at the end. One of the most significant engineering polymers is polyethylene terephthalate (PET), which is widely used. Due to its numerous crucial qualities, it has a wide variety of applications, from packaging to fibers. The thermoplastic polyolefin, primarily polyethylene and polypropylene (PP), is a popular choice utilized globally in a wide range of applications. In the first phase of the current experiment, the materials were obtained by hot pressing with the press machine. The reinforcer is made of Al nanopowder 800 nm and Fe nanopowder 790 nm and the quality of the recycled polymer was examined using Fourier transform infrared spectroscopy (FTIR), a scanning electron microscope (SEM), and differential scanning calorimetry (DSC). From DSC variation curves as a function of temperature, the values from the transformation processes (glass transition, crystallization, and melting) are obtained. SEM measurements revealed that the polymer composites with Al have smooth spherical particles while the ones with Fe have bigger rough spherical particles.

## 1. Introduction

As part of the EU’s circular economy action plan, the European Commission adopted a European strategy for plastics in 2018 [1]. The goal was to support, improve, and accelerate the implementation of measures to reduce plastic waste. Another significant objective is the requirement that all plastic packaging sold in the EU by 2030 either be reusable or can be recycled economically [2]. The question of how the European Union’s strategies for plastics in a circular economy will affect food safety and whether any associated risks to food safety can be identified and quantified arises when closed-loop recycling, also known as recycling food packaging into new materials for food packaging, is also on this agenda [3]. The packaging industry faces significant obstacles in recycling packaging polymers as a result of this stringent EU policy. In fact, almost all significant packaging, food, and industrial associations have published their re-collection and recycling goals for the years after 2025 [4]. Plastics have evolved into an indispensable component of our contemporary society in the twenty-first century. It is one of the most widely used commodities in daily life, with applications in furniture, automotive components, packaging, body implants, aviation, and aerospace. Plastics are so popular because they have a broad variety of features and can even be customized to have specific properties [5]. Plastics are strong, long-lasting, moisture-resistant, and non-biodegradable materials that make up approximately 80% of solid garbage gathered in landfills, municipal trash dumps, and other locations [6,7]. Due to its significant contribution to garbage, plastic waste has been divided into primary and secondary scrap. Despite being so versatile, plastic is not a preferred material because of the annoyance it causes [8,9]. These primary and secondary trashes, which are resistant to degrading processes, build up in the environment and endanger life on land and in water. Environmentalists’ attention has been drawn to the resistance of polymers, particularly in the cases of polyethylene, polystyrene, polyethylene terephthalate (PET), or polypropylene (PP), in order to develop an idea for appropriate disposal methods that are safer and more advantageous for society [10,11]. Choosing one of them, PET, a semi-aromatic thermoplastic co-polymer resin from the polyester family, will be the subject of this discussion [12,13]. PET has the following characteristics: High strength, decreased density, resistance to physical and chemical degradation, low gas permeability, and non-biodegradable compound [14,15]. PET pollution is now managed using a variety of techniques, including mechanical, thermal, and chemical-based [16,17]. Even though mechanical recycling is the most popular approach, it has limits since only surface-level contaminants are eliminated. The volatile organic compounds that moved to the polymer matrix remained there as a result [18,19]. Chemical recycling is based on depolymerization, whereas mechanical recycling includes melting. Chemical recycling is being utilized in a variety of industrial and business settings, including those that include interaction with food [20,21,22,23]. According to the literature, many analytical techniques have been used to quantify surrogate pollutants in contaminated and recycled PET [24,25]. Recycling both mono- and mixed-plastic materials presents distinct challenges. The main problem is that polymers will break down under certain conditions. Heat, oxidation, light, ionic radiation, hydrolysis, and mechanical shear are among these conditions). During the mechanical reuse of polymers, two sorts of degradation occur: Debasement caused by going back over (warm mechanical debasement) and degradation during lifetime. During melt processing, the heating and mechanical shearing of the polymer causes thermal-mechanical degradation [26,27].

The recycling of materials is frequently categorized within the framework of the circular economy based on the product that is produced from the secondary raw materials: In closed-loop recycling when the same kind of product from which the recycled plastics were originally recovered is produced using them [28,29] and in open-loop recycling when a different kind of product from which the recycled plastics were originally recovered is produced using them [30,31].

The shape of metal oxide nanoparticles, which results from the production process, is intimately related to their characteristics. With a focus on bio-nanocomposites, commonly used metal oxides, and potential mixed metal or doped metal oxides, a recent review presents current innovative synthesis methods for producing metal oxide nanoparticles and current incorporation techniques used to produce smart (active and/or intelligent) packaging [32]. Other authors reviewed the most recent advancements in the use of metallic-based micro- and nanocomposites in food packaging solutions while taking into consideration the restrictions set out by food laws [33]. Metal scavengers make up the largest market group and have been employed in commerce for a long time. The most often used agents for the preservation of packaged goods are oxygen scavengers based on iron. These systems’ great efficacy, low cost, and quick rate of oxidation are the reasons they are successful commercially [34].

When the polymer is subjected to both temperature and shear, a variety of processes will begin. Chain scission and chain branching are the most prevalent mechanisms found in commercial polymers. Thermal properties (melting temperature, crystallization, etc.) in addition to the variation in mechanical and rheological properties and physical characteristics (such as color, surface properties, etc.) and thermal mechanical degradation has an effect. By including various additives, these thermal-mechanical degradation effects can be mitigated. By including various additives, these thermal-mechanical degradation effects can be mitigated [35].

Light-sensitive substances are often packaged in dark-colored vials to protect them from UV radiation and light. The disadvantage is that, in this case, the original color of the preparation is no longer visible, and the medicine cannot be checked for particles or color changes before dispensing it. While clear containers allow inspection of the liquids they contain, they allow light and UV rays to pass through plastics/glass. A remedy would be to use a clear inspection window with UV protection or a resealable inspection window that protects against UV rays and blue light. Manufacturers of biologics and other sensitive products are thus able to effectively protect their highly sensitive substances against light and UV irradiation, avoiding potential health risks for patients from drugs that have been damaged by light.

The advantages of thermoplasticity, associated with inserts of metal particles, make such composites easy to process technologically, with minimal costs and resource saving, in the sense of reducing the amount of silver or other protective ingredients in the current packaging. On the other hand, the developed packaging can be used in a cascade system and is directly printable, which gives it a great economic and aesthetic advantage on the market.

In particular, blister packaging is gaining popularity due to its ease of use as a unit-dose packaging system. This packaging requires fewer resources to manufacture, provides compact packaging, has a longer shelf life, better product visibility, easy handling and availability at lower costs compared to rigid bottles, pouches, etc. The growing sale of over-the-counter medicines and the increased level of product safety against contaminants such as moisture and oxygen are also driving the growth of this type of packaging. This type of packaging is easier to use and can also be customized according to the customer’s requirements.

In this context, the work aims to develop new types of packaging from recycled materials and to study their physico-chemical properties. In order to improve mechanical and barrier qualities, inhibit the photodegradation of plastics, and improve food contact polymer performance, metallic-based materials are added to these materials.

## 2. Materials and Methods

### 2.1. Materials

Polyethylene terephthalate came from SC ALL GREEN SRL Iasi from its own recycling sources, polypropylene Tipplen H 318 (Ic = 12), high-density polyethylene Tipelin 1100 J, (Ic = 7.5), with a degree of crystallinity of 71%.

The reinforcer is made of Al nanopowder 800 nm and Fe nanopowder 790 nm from the company NANOGRAFI LTD.STI, Ankara Turkey.

**Figure 1:**
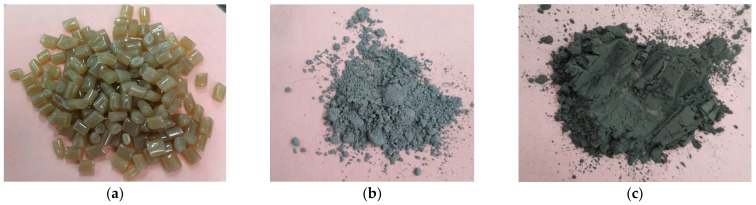
(**a**) Recycled PET granules, (**b**) Al nanopowder, and (**c**) Fe nanopowder.

Figure 1 shows the raw materials used to obtain the composite materials.

### 2.2. Sample Preparation

In the first phase, we attempted to obtain the materials by hot pressing with a press machine at a temperature of 310 °C.

**Figure 2:**
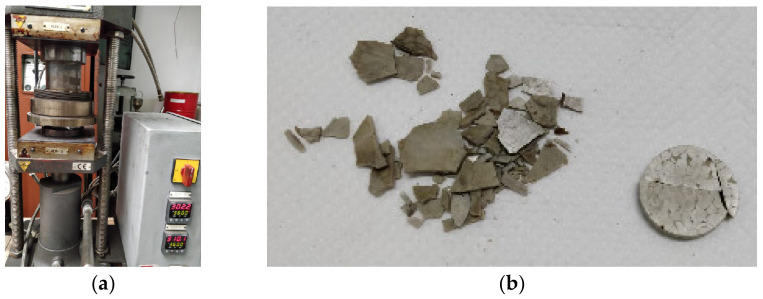
(**a**) Press with hot plates; (**b**) the resulting plates.

Very friable pieces were obtained that could not be extracted whole from the mold. The press and the resulting plates are presented in Figure 2.

These composite materials can be classified into 3 sets:

- 5 experimental models of composite materials using a PET polymer matrix and Al and Fe nanopowders reinforcers of 800 nm in grain size in concentrations of 5% and 8%, codified as follows:
  ○ M1 recycled PET.
  ○ M2 PET + 5% Al nanopowder.
  ○ M3 PET + 8% Al nanopowder.
  ○ M4 PET + 5% Fe nanopowder.
  ○ M5 PET + 8% Fe nanopowder.
- 5 experimental models of composite materials using as a polymer matrix a mixture of PET and PP polymers and Al and Fe nanopowder reinforcers of 800 nm granulation in a concentration of 5% and 8%, codified as follows:
  ○ M6 PET 70% + PP 30%.
  ○ M7 PET + PP + 5% Al nanopowder.
  ○ M8 PET + PP + 8% Al nanopowder.
  ○ M9 PET + PP + 5% Fe nanopowder.
  ○ M10 PET + PP + 8% Fe nanopowder.
- 5 experimental models of composite materials using as a polymer matrix a mixture of two polymers PET + HDPE (High-Density Polyethylene) and reinforcing Al and Fe nanopowders of 800 nm granulation in a concentration of 5% and 8%, coded as follows:
  ○ M11 PET 70% + HDPE 30%.
  ○ M12 PET + HDPE + 5% Al nanopowder.
  ○ M13 PET + HDPE + 8% Al nanopowder.
  ○ M14 PET + HDPE + 5% Fe nanopowder.
  ○ M15 PET + HDPE + 8% Fe nanopowder.

**Figure 3:**
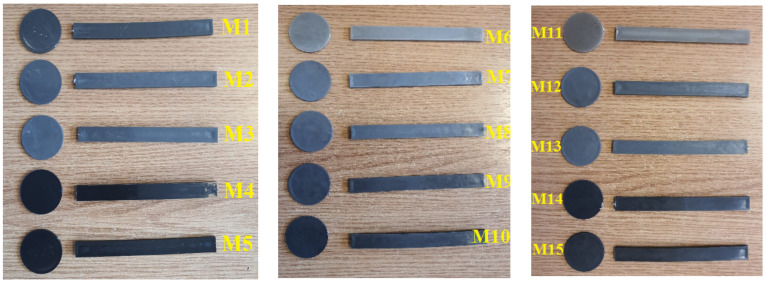
Experimental models of composite materials, coded M1–M15.

The composite materials obtained from this set are shown in Figure 3, while the processing temperature regimes presented by the machine interface are presented in Table 1.

It is found that composite materials containing Al nanopowders (M2, M3, M7, M8, M12, and M13) are easy to process compared to those containing Fe nanopowders (M4, M5, M9, M10, M14, and M15) due to the fact that aluminum is a soft metal that allows easy embedding in the polymer matrix, which also induces better homogenization.

To remedy this shortcoming, it is recommended to process composite materials through the two classical stages: The extrusion that performs the mixing of the polymeric material with the reinforcer (the nanopowder) and then injection from the melt into the shapes required for the final packaging. Through these two processing stages, good homogenization is obtained, but it involves higher costs.

### 2.3. Characterization Methods

For the optical characterization of the samples of thermoplastic packaging received from the beneficiary, an FTIR spectrophotometer, model Vertex 80, from Bruker was used. These spectra were recorded in the totally attenuated reflection geometry (ATR). The spectra were recorded using the OPUS 6.0 acquisition program. According to the protocol, it was necessary to produce a background and then record each test sample. The parameters used were as follows: The spectral range was 600–4000 cm^−1^, the number of scans was 64, and the resolution was 4 cm^−1^. After recording each spectrum, atmospheric compensation was performed in order to eliminate the contribution of water vapor and CO_2_. The spectra were saved with the dpt. extension, being imported into Origin 2018.

SEM scanning optical microscopy analysis was performed with a Scanning Electron Microscope with a field emission source and focused ion beam. Images were taken at an accelerating voltage of 1 or 2 kV with very close proximity to the objective lens. The detector used was the Everhart Thornley type secondary electron detector with a Faraday cup—resulting in micrographs that highlight the morphology and topography of the analyzed surfaces. The fields recorded in these micrographs are relatively narrow, from a few hundred microns to 10–20 microns, depending on the magnification used, with the analyzed materials not showing major variations in two randomly analyzed fields.

Thermogravimetric analysis and differential calorimetry (TGA/DSC) were carried out with the help of the simultaneous thermal analyzer TGA-DSC type STA 449 F3 Jupiter, NETZSCH, Germany. The conditions of the TGA/DSC measurements performed on solid samples of composite polymer materials (5–10 mg) were as follows: A temperature range of 25–300 °C; a heating speed of 10 K/min; a working atmosphere of nitrogen; a reference substance of aluminum crucible. Before introducing the sample to be analyzed into the device, it was weighed on a digital balance type Precisa XT 220A (Switzerland), with a digital display with a precision class of 0.1 mg.

## 3. Results and Discussions

### 3.1. FTIR Measumeremts

**Figure 4:**
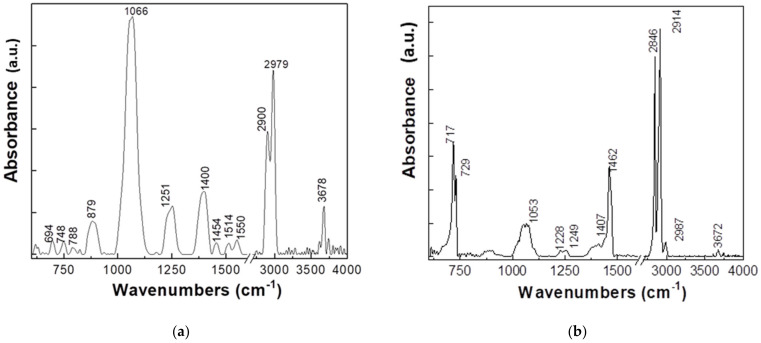

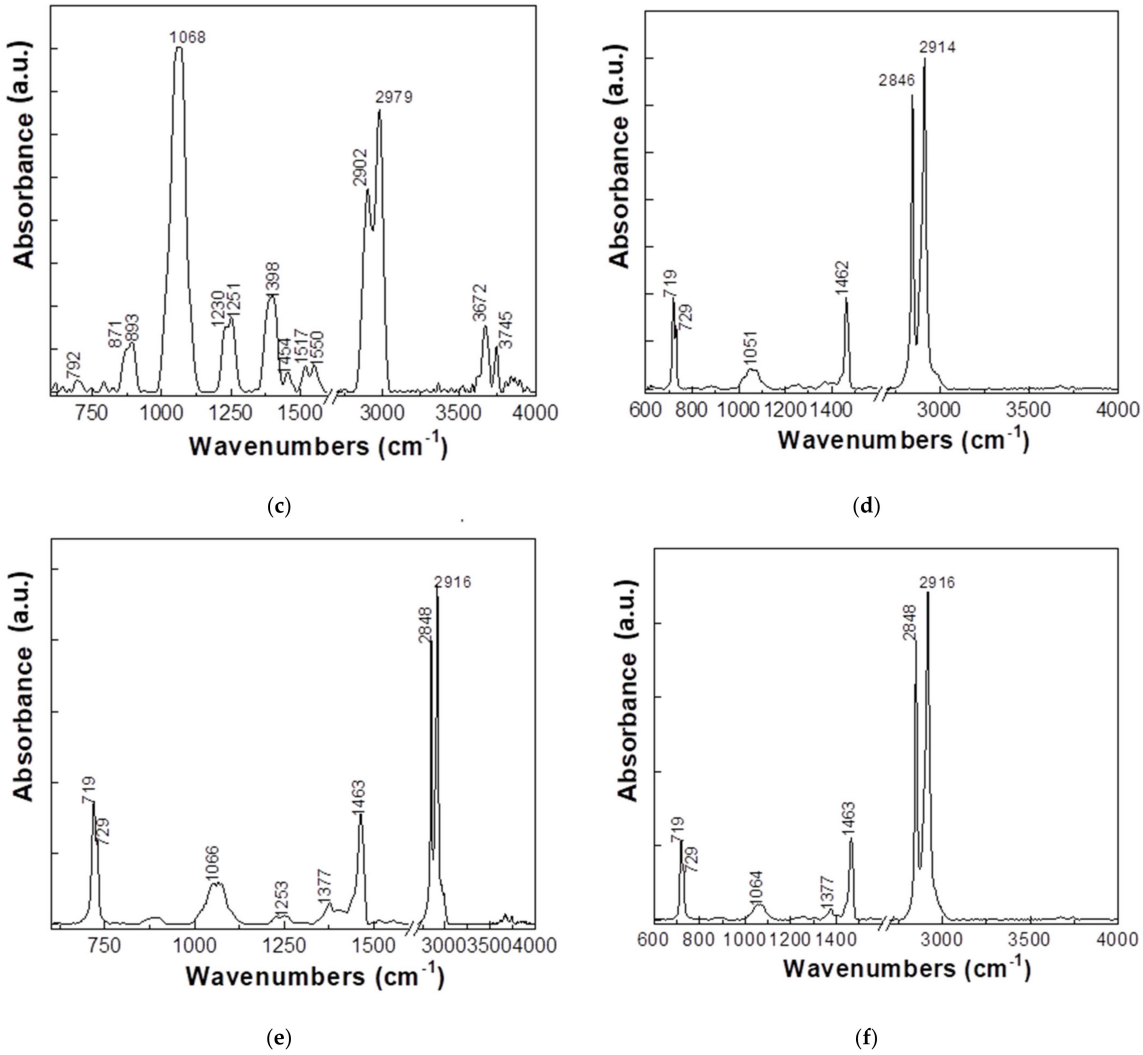

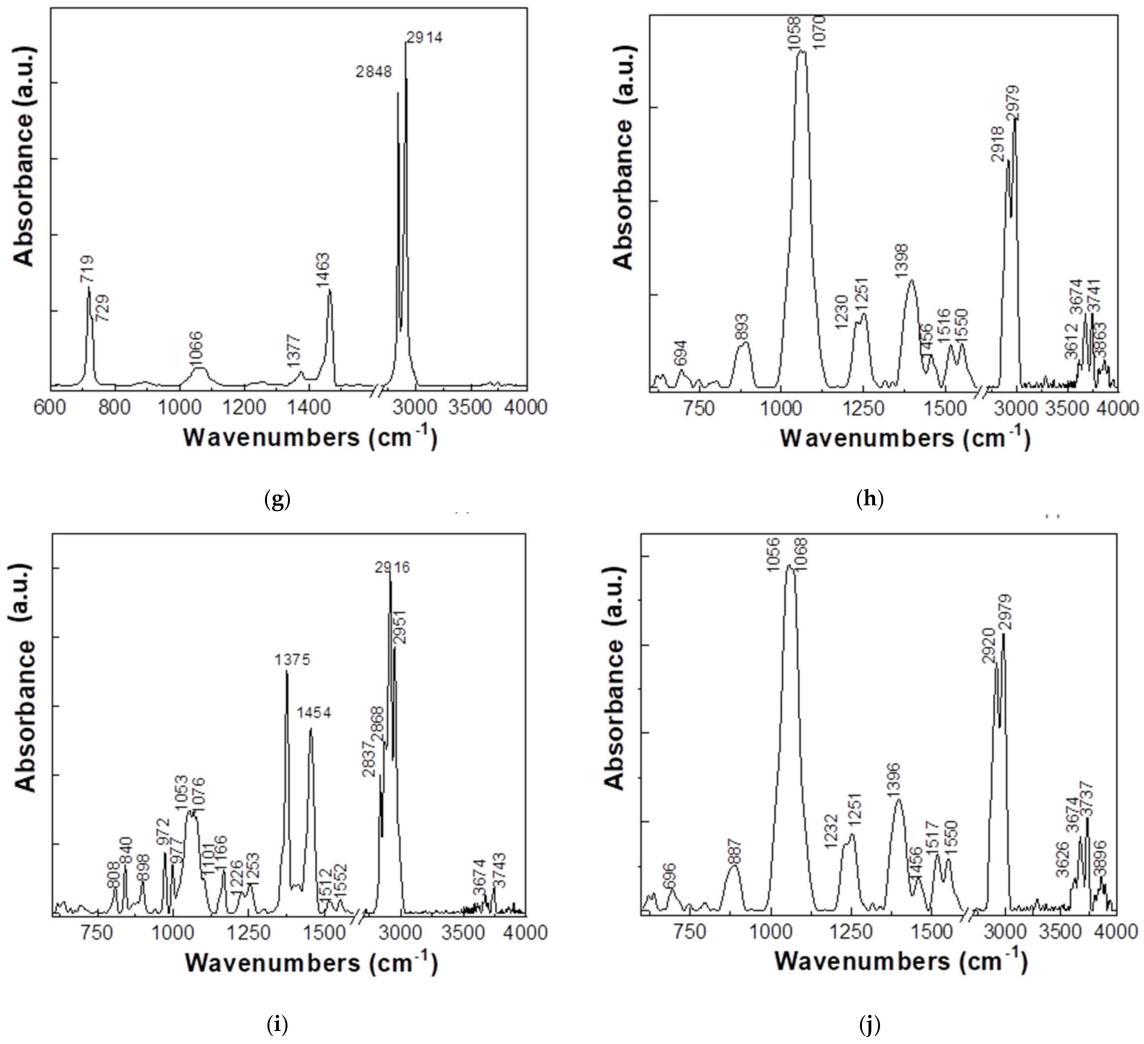
FTIR spectra of the labeled samples by SC All Green SRL as follows: (**a**) HDPE + 8% Fe, 50 nm; (**b**) HDPE + 8% Al, 50 nm; (**c**) HDPE + 5% Al, 800 nm; (**d**) HDPE + 3% Ferrite; (**e**) LDPE + 5% Fe, 50 µm; (**f**) LDPE + 5% Ferrite; (**g**) LDPE + 8% Al; (**h**) PP + 8% Ferrite; (**i**) PP + 8% Fe 800 nm and (**j**) PP + 8% Al, 50 nm.

FTIR absorption spectra are illustrated in Figure 4.

The analysis of the FTIR spectra presented in Figure 4 shows the case of the labeled sample as follows:

(a) HDPE + 8% Fe, 50 nm, has two IR bands with higher absorbance at approximately 1066 and 2900–2979 cm^−1^ being accompanied by other IR bands with lower absorbance having maxima at 879, 1251, 1400, 1454, 1515–1550, and 3678 cm^−1^.
(b) HDPE + 8% Al, 50 nm, presents the following IR bands with maxima at 717, 729, 1053, 1462, 2846, and 2914 cm^−1^.
(c) HDPE + 5% Al, 800 nm, shows two intense bands at 1068 and 2902–2979 cm^−1^, being accompanied by other IR bands with lower absorbance at approximately 871–893, 1230–1251, 1398, 1454, 1517–1550, and 3672–3745 cm^−1^.
(d) HDPE + 3% Ferrite presents the following IR bands with maxima at approximately 719–729, 1051, 1462, and 2846–2914 cm^−1^.
(e) LDPE + 5% Fe, 50 µm, presents IR bands with maxima at approximately 719–729, 1066, 1463, and 2848–2916 cm^−1^.
(f) LDPE + 5% Ferrite, the position of the main IR bands is approximately 719–729, 1064, 1463, and 2848–2916 cm^−1^.
(g) LDPE + 8% Al, presents IR bands with maxima at approximately 719–729, 1066, 1463, and 2848–2914 cm^−1^.
(h) PP + 8% Ferrite, presents IR bands with maxima at approximately 893, 1058–1070, 1230–1251, 1398, 1454, 1516–1550, 2918–2979, and 3612–3674–3741 cm^−1^.
(i) PP + 8% Fe, 800 nm, shows IR bands with maxima at approximately 808, 840, 898, 977, 1101, 1053–1076, 1166, 1226–1253, 1375, 1454, 2837–2868–2916–2951, and 3674–3743 cm^−1^.
(j) PP + 8% Al, 50 nm, presents IR bands with maxima at 887, 1056–1068, 1232–1251, 1396, 1454, 1517–1550, 2920–2979, and 3626–3674–3737 cm^−1^.

HDPE + 8% Al, 50 nm, HDPE + 3% Ferrite, LDPE + 5% Fe, 50 µm, LDPE + 5% Ferrite, and LDPE + 8% Al samples show IR bands that are located in the vicinity of those reported in the case of polyethylene at approximately 720–731, 1463, 2851, and 2919 cm^−1^; they were attributed to the vibrational modes of tilting deformation, bond deformation, symmetric stretching of the CH_2_ bond, and asymmetric stretching of the CH_2_ bond, respectively [36].

The sample PP + 8% Fe, 800 nm shows IR bands whose maxima are in the vicinity of those located at approximately 992, 997, 1375, 1452, 2839, 2872, 2916, and 2953 cm^−1^. They were assigned as follows: The first two vibrational modes related to the isotactic entities of the macromolecular chains and the bond and stretching vibrational modes of the methylene group, respectively [37,38,39].

### 3.2. DSC Measurements

**Figure 5:**
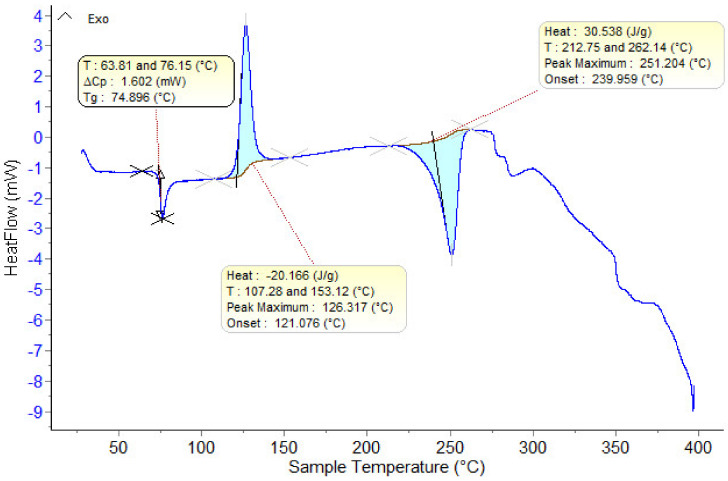
DSC-recorded curve on the original PET sample.

In Figure 5, the DSC curve for the initial (undried) PET is shown.

The material presents the following thermal effects: A glass transition temperature (Tg) at approximately 75 °C; a thermal crystallization temperature at approximately 126 °C; a melting temperature at approximately 251 °C.

**Figure 6:**
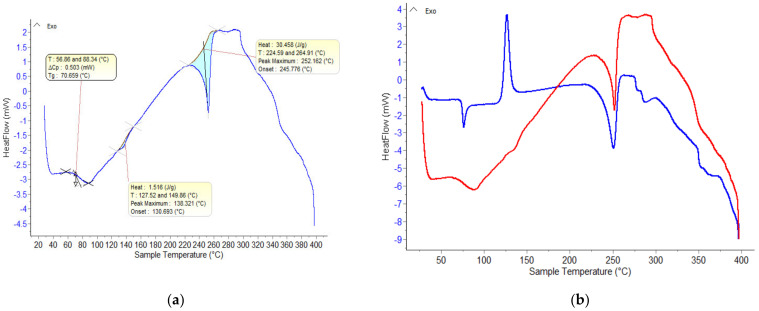
(**a**) DSC curve recorded on the PET sample subjected to heat treatment (drying at 100 degrees for 8 h); (**b**) comparative DSC curves: initial PET (blue) and PET subjected to heat treatment (red).

From Figure 6, the thermally treated material shows the following thermal effects: A glass transition temperature (Tg) at approximately 70 °C and thermal crystallization temperature is absent. This is caused by heating the material above Tg, followed by its slow cooling. The material becomes opaque due to the spherulitic structure formed by the thermally induced crystalline aggregates of the non-oriented PET chains. Rapid cooling of the sample would have led to obtaining a material with a high degree of amorphousness, which subsequently, upon reheating, would have presented the phenomenon of thermal crystallization noticed in Figure 6 with a melting temperature of approximately 252 °C.

**Figure 7:**
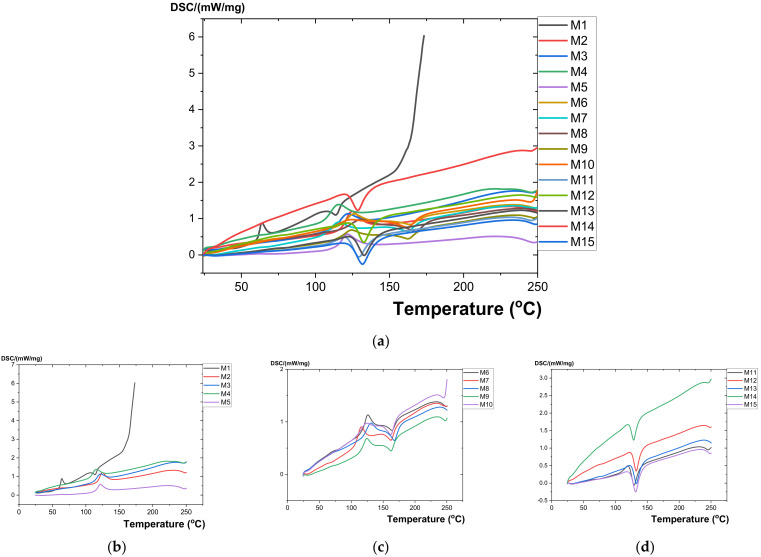
(**a**) DSC variation curve for all samples (M1–M15); (**b**) DSC variation curve for M1–M5 (M1 is recycled PET); (**c**) DSC variation curve for M6–M10; (**d**) DSC variation curve for M11–M15.

Figure 7 illustrates the analyses of the DSC variation curves as a function of temperature (25–300 °C) presented for all studied composite materials (M1–M15).

The composite materials were obtained by injection from the melt on the injection machine Dr. Boy, Germany. Thus, the experimental models are presented in Table 2.

In Table 2, the resulting values from the transformation processes (glass transition, crystallization, and melting) are presented, which were obtained from the analysis of the DSC variation curves as a function of temperature.

The composite materials thermally analyzed with the TGA/DSC analyzer consisted of a basic matrix of polyethylene terephthalate (recycled PET—M1) to which the following were added (Table 3): Reinforcement elements consisting of nanometric metal powders (800 nm) of Al (M2, M3); reinforcing elements consisting of nanometric metal powders (800 nm) of Fe (M4, M5); polypropylene Tipplen H 318-PP) (M6); polypropylene Tipplen H 318–PP and nanometric metal powders (800 nm) from Al (M7, M8); polypropylene Tipplen H 318–PP and nanometric metal powders (800 nm) from Fe (M9, M10); high-density polyethylene Tipelin—HDPE (M11); Tipelin high-density polyethylene—HDPE and nanometric metal powders (800 nm) from Al (M12, M13); Tipelin high-density polyethylene—HDPE and nanometric metal powders (800 nm) from Fe (M14, M15).

Following thermal analysis, it was found that all the studied composite materials (M1 … M15) had no mass loss transformations, which means that these materials can be used at up to 300 °C. All studied polymeric materials (M1 … M15) underwent a glass transition process (second-order phase transition) when the rubbery state changed to the glassy solid state. That happens with a transformation start temperature in the range of 63 … 74 °C and a variation of ΔCp in the range of 0.001 … 0.076 J/gK. This process occurs due to the presence of amorphous areas in the analyzed samples.

Both for the polymer material made of polyethylene terephthalate (recycled PET—M1) and the polymer composite materials with a matrix of polyethylene terephthalate (recycled PET), to which reinforcement elements consisting of nanometric metal powders (800 nm) of Al (M2, M3) or Fe (M4, M5) were added, the existence of three thermal processes was found: Glass transition, crystallization, and melting (Figure 7).

Both for the composite polymer material made of polyethylene terephthalate (recycled PET—M1) and polypropylene Tipplen H 318-PP) (M6), as well as for the polymer composite materials with a matrix of polyethylene terephthalate and polypropylene Tipplen H 318, to which elements of reinforcement consisting of nanometric metal powders (800 nm) of Al (M7, M8) or Fe (M9, M10) were added, the existence of a glass transition thermal process, a thermal crystallization process, and two melting processes was found.

For the composite polymer material made of polyethylene terephthalate (recycled PET—M1) and Tipelin high-density polyethylene—HDPE (M11), as well as for the polymer composite materials with a matrix of polyethylene terephthalate and Tipelin high-density polyethylene, to which reinforcing elements consisting of nanometric metallic powders (800 nm) of Al (M12, M13) or Fe (M14, M15) were added, the existence of a glass transition thermal process and two melting processes was found.

### 3.3. Measurements of Hydrostatic Density

The hydrostatic density is determined with the Metler Toledo Analytical Balance, which has the following characteristics: A maximum capacity of 220 g; precision of 0.1 mg; linearity of ±0.2 mg; internal calibration; density kit for solids and liquids; and interface RS 232.

The maximum resolution of hydrostatic density measurements in ethanol at 20 °C is 0.0005 g/cm^3^ (Table 4). The measurements were performed at 21 °C with three consecutive repetitions and the error was calculated. The density was determined as the mean value between three consecutive repeated measurements.

From the experimental results obtained for the hydrostatic density, it can be seen that the values are close, a fact justified by the majority content in the polymer (the polymer concentrations used were 100%, 95%, and 92%, respectively).

The composite material M10 has the highest density and M6 has the lowest compared to the other composite materials obtained.

The swelling capacity of polymers is determined by the amount of liquid that the material can absorb when immersed in a liquid. In the case of this report, water was chosen as the liquid swelling medium as a universal solvent for packaging.

### 3.4. Measurements of Swelling Capacity

To determine the swelling capacity in water for the composite materials studied, the procedure was carried out according to SR EN ISO 175/2011 [40]: approximately 0.085 g of composite material was weighed on average and placed in plastic ampoules with tight caps (tubes for micro-Centrifuges with a diameter of 10 mm and a length of 40 mm); vials with composite material, thus made, were filled with deionized water and maintained for 72 h at a temperature of 22 °C (atmospheric) and a humidity of 41%.

To determine the degree of swelling, Formula (1) was used:

(1) $$Q=\frac{X_{2}-X_{1}}{X_{1}}\times 100$$

where:

*Q*—degree of swelling.
*X*_2,3,4,…_—mass of swollen polymer (after each 168-h cycle).
*X*_1_—dry polymer mass.

An analytical balance was used to determine the mass variation (Table 5).

**Figure 8:**
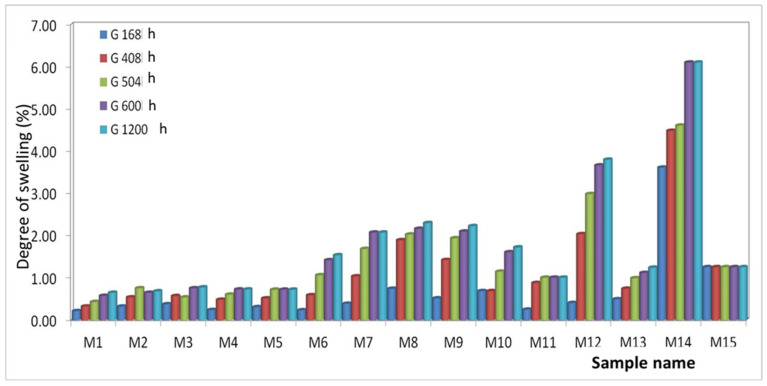
Water swelling of materials M1-M15.

The swelling times in water were 168, 408, 504, 600, and 1200 h (Figure 8).

From the experimental results, the following classification of the composite materials studied can be made from the point of view of the increase in the degree of swelling in water depending on the exposure time.

After 168 h for polymer matrix materials from:

- PET (M1–M5): It is found that the highest water absorption is presented by M3 and the lowest by M1. This can be justified by the fact that the percentage of 8% Al nanopowder creates the most voids in the mixing process in the melt, which creates the possibility of inserting water. The values of the degree of swelling are very close due to the majority concentration of the polymer. The classification of materials is as follows: Q M1 < Q M4 < Q M5 < Q M2 < Q M3;
- PET + PP (M6–M10): It is found that M8 has the highest water absorption and M1 has the lowest. Here it can be seen that the Al nanopowder creates more voids than the Fe one. The classification of materials is as follows: Q M6 < Q M7 < Q M9 < Q M10 < Q M8;
- PET + HDPE (M11–M15): It is found that M14 has the highest water absorption and M11 has the lowest. In this case, it is observed that the materials containing Al nanopowder have a lower degree of swelling than those with Fe nanopowder. It can be said that in the PET + HDPE polymer mixture, Al nanopowder is better homogenized than Fe. The classification of materials is as follows: Q M11 < Q M12 < Q M13 < Q M15 < Q M14;

After 408, 504, 600, and 1200 h, it was found that the direction of the increase in the degree of swelling is the same as after 168 h.

It can be said that the studied materials are resistant to the action of water. The most hygroscopic material among those analyzed is M14.

### 3.5. SEM Measurements

**Figure 9:**
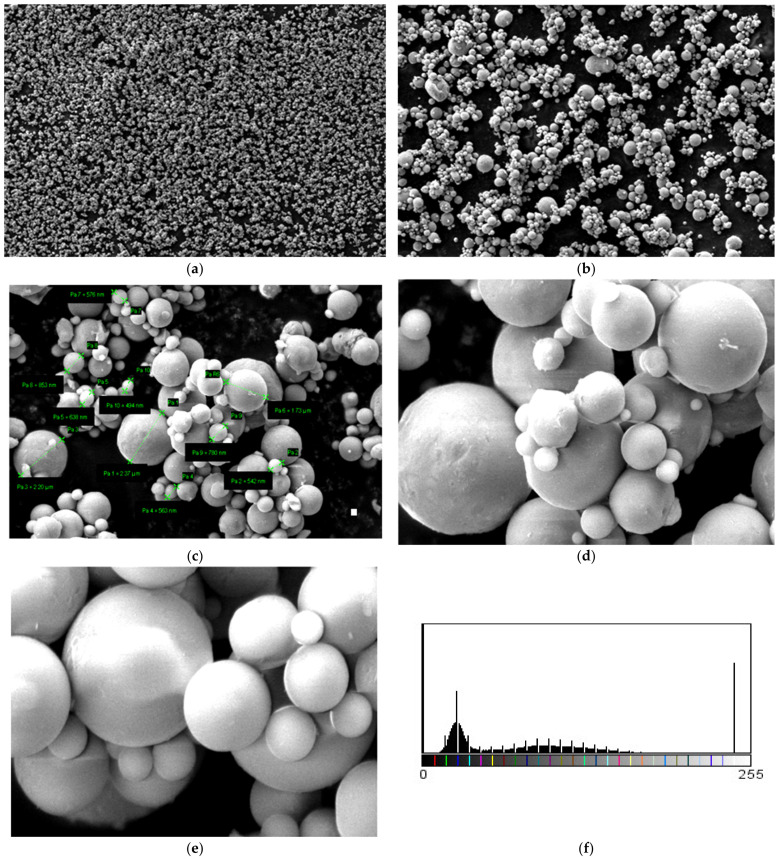
Micrographs for Al powder 800 nm (**a**) 1000×, (**b**) 5000× (**c**) 20,000×, (**d**) 50,000× (**e**) 100,000×, and (**f**) histogram of Al nanoparticles (pixels).

Micrographs were made for Al powder and are shown in Figure 9. A large dispersion of particle sizes is observed from the micrographs. To highlight this, 10 measurements of Al 800 nm powder particles were made, and the following values were observed: 2.37 μm, 542 nm, 638 nm, 1.73 μm, 576 nm, 853 nm, 780 nm, and 494 nm. It can be said that this nanopowder has smooth spherical particles and an average size of 798 nm.

**Figure 10:**
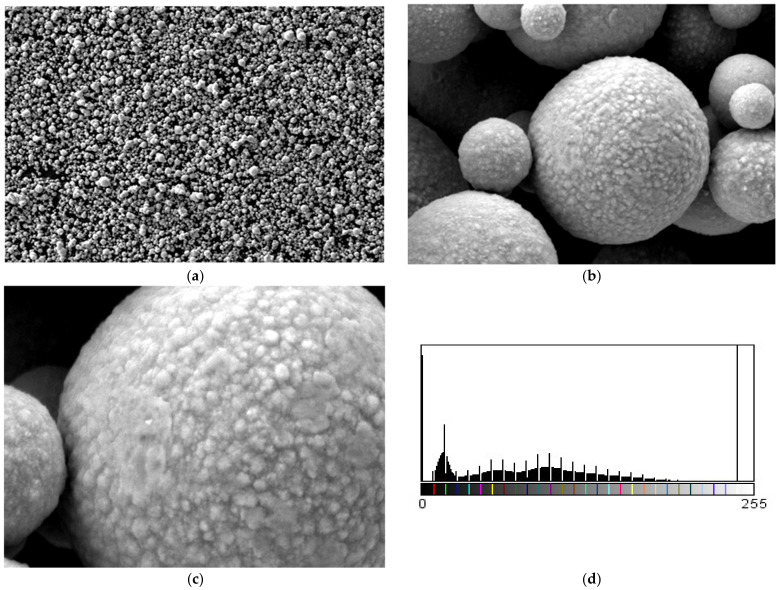
Micrographs for Fe nanopowder 800 nm (**a**) 20,000×, (**b**) 50,000×, (**c**) 100,000×, and (**d**) histogram of Fe nanoparticles (pixels).

For the 800 nm Fe nanopowder, micrographs are shown in Figure 10. A large dispersion of particle sizes is found. The evidence of the differences in the granulation of the Fe 800 nm powder is presented as the results of six measurements of the powder particles and the following values were observed: 7.25 µm, 2.9 µm, 3.01 µm, 1.01 µm, 769 nm, and 1.23 µm. It can be said that this nanopowder has rough spherical particles with an average size of 2.78 µm.

**Figure 11:**
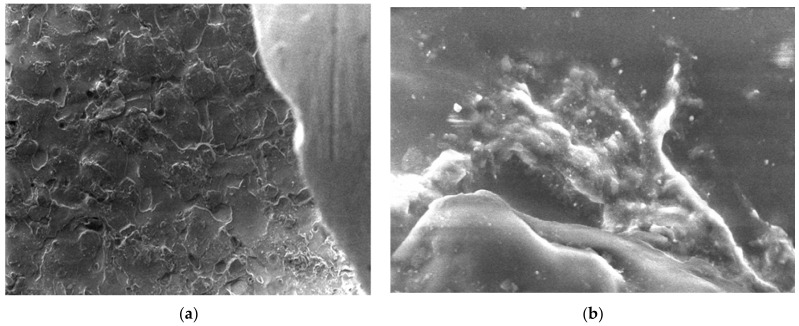
Micrographs for PP at (**a**) 500× and (**b**) 20,000× magnifications.

In the case of polypropylene (PP), which is a natural polymer, the structure of the polymer and the melting process can be observed from the micrographs. For this material, micrographs were formulated. These images are presented in Figure 11.

For the obtained composite materials, micrographs were made at magnifications of 500 and 50,000 as shown in Figure 12, Figure 13, Figure 14, Figure 15, Figure 16, Figure 17, Figure 18, Figure 19, Figure 20, Figure 21, Figure 22 and Figure 23. 

From the micrographs produced on the obtained composite materials, it can be said that:

- It can be observed from the composition that for M7 and M8, the smooth powder particles are specific to aluminum, and for M8 and M10, the rough spherical particles are specific to iron.
- Among these experimental models, it is found that the most homogeneous is M7 (the agglomerations are less), making it optimal for the production of special packaging to be used in the food and pharmaceutical industries.

## 4. Conclusions

The development of novel metallic-based nanocomposites containing metal-loaded inorganic materials or metal nanoparticles is providing advanced properties for tailored applications, which are being explored in food packaging. Although they typically enhance membrane function, metallic nanoparticles have the potential to alter or even degrade membrane performance.

To identify the best kinds and compositions of nanoparticles to include in polymeric membranes, considerable research must be performed. In this study, FTIR, SEM, and DSC methods were chosen to study the pristine and composite materials. The composite material PET + PP + 8% Fe nanopowder has the highest density and PET 70% + PP 30% has the lowest compared to the other composite materials obtained. The polymer composites containing Al nanopowder have smooth, spherical particles, whereas the ones containing Fe have rough, spherical bigger particles, as determined by SEM measurements. The studied materials are resistant to the action of water. The most hygroscopic material among those analyzed is PET + HDPE +5% Fe nanopowder.

However, laws must take into account possible dangers related to nano-dimensions and the probable migration of metal ions into foods and beverages prior to commercial application.

## Figures and Tables

**Figure 12:**
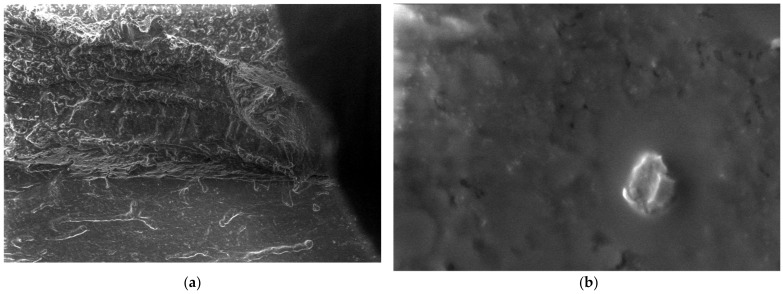
Micrographs for the M1 composite material at (**a**) 500× and (**b**) 50,000× magnifications.

**Figure 13:**
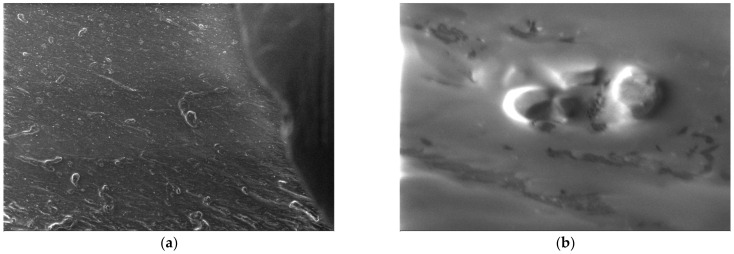
Micrographs for the M2 composite material at (**a**) 500× and (**b**) 50,000× magnifications.

**Figure 14:**
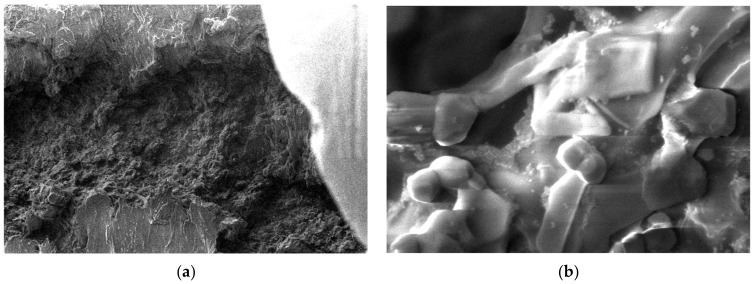
Micrographs for the M3 composite material at (**a**) 500× and (**b**) 50,000× magnifications.

**Figure 15:**
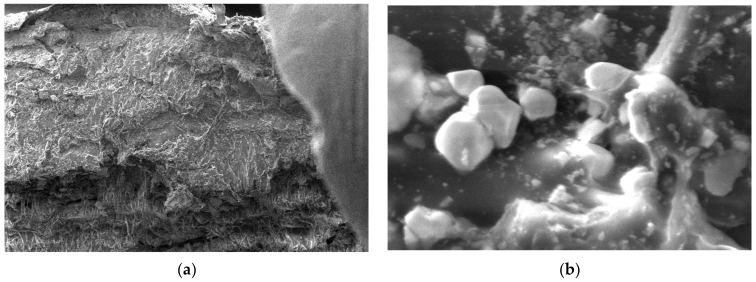
Micrographs for the M4 composite material at (**a**) 500× and (**b**) 50,000× magnifications.

**Figure 16:**
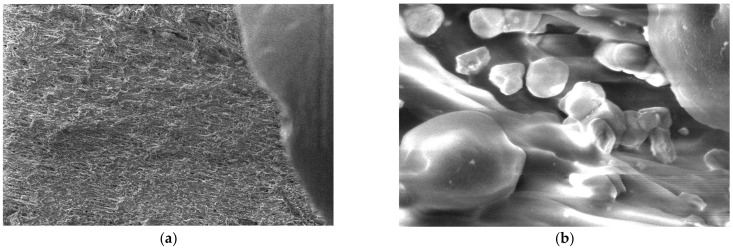
Micrographs for the M5 composite material at (**a**) 500× and (**b**) 50,000× magnifications.

**Figure 17:**
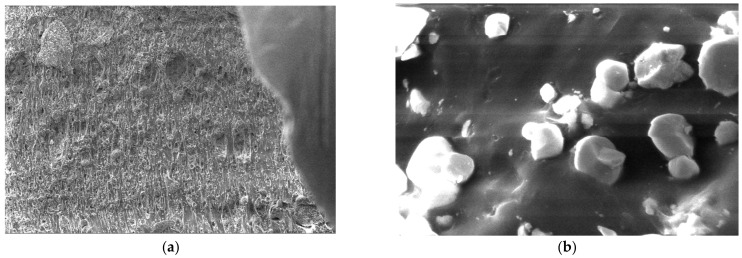
Micrographs for the M6 composite material at (**a**) 500× and (**b**) 50,000× magnifications.

**Figure 18:**
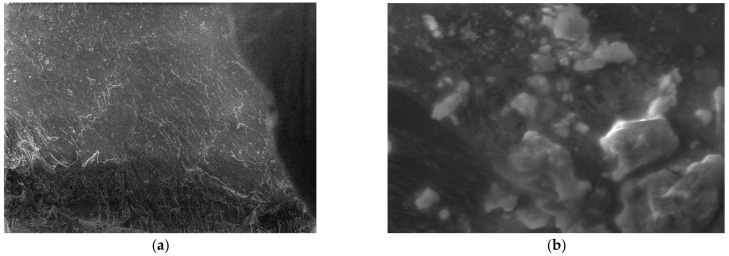
Micrographs for the M7 composite material at (**a**) 500× and (b) 50,000× magnifications.

**Figure 19:**
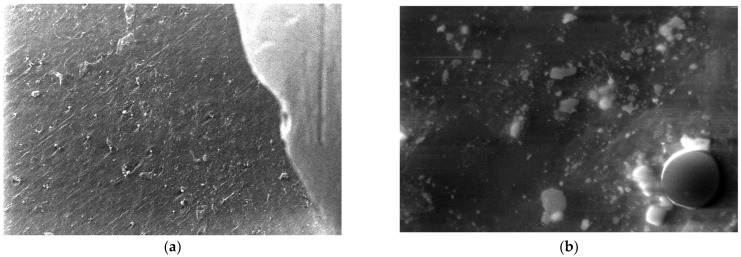
Micrographs for the M8 composite material at (**a**) 500× and (**b**) 50,000× magnifications.

**Figure 20:**
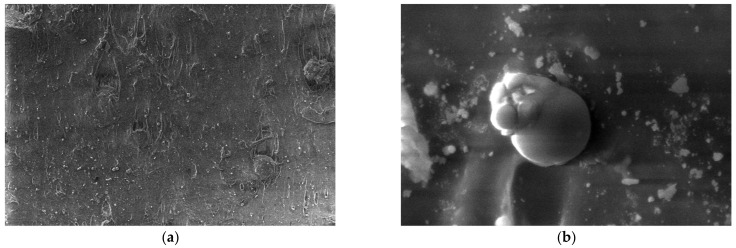
Micrographs for the M9 composite material at (**a**) 500× and (**b**) 50,000× magnifications.

**Figure 21:**
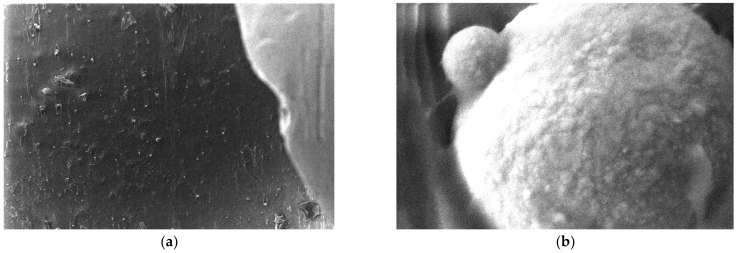
Micrographs for the M10 composite material at (**a**) 500× and (**b**) 50,000× magnifications.

**Figure 22:**
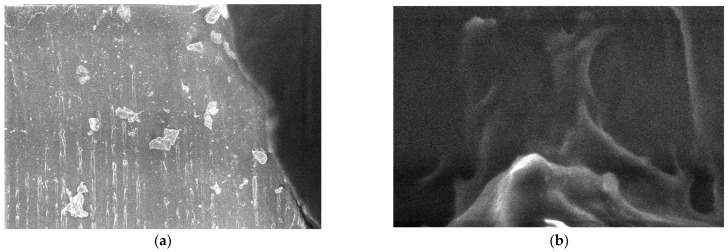
Micrographs for the M11 composite material at (**a**) 500× and (**b**) 50,000× magnifications.

**Figure 23:**
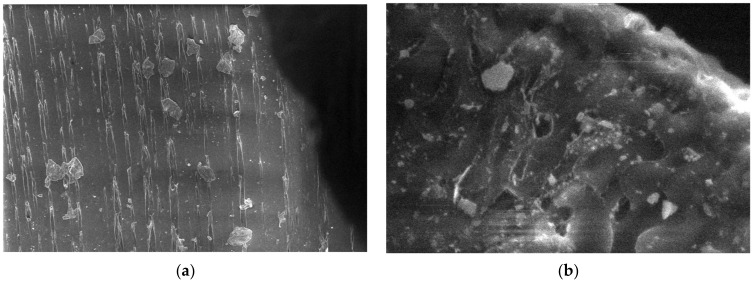
Micrographs for the M12 composite material at (**a**) 500× and (**b**) 50,000× magnifications.

**Table 1:** The processing temperature regimes presented by machine interface for M1–M15.

Encode	The Temperatures in the Heating Zones (°C)				
M1	300	295	290	285	280
M2	260	255	250	245	240
M3	260	255	250	245	240
M4	270	265	260	255	250
M5	270	265	260	255	250
M6	260	255	250	245	240
M7	260	255	250	245	240
M8	260	255	250	245	240
M9	260	255	250	245	240
M10	260	255	250	245	240
M11	260	255	250	245	240
M12	250	245	240	235	230
M13	250	245	240	235	230
M14	250	245	240	235	230
M15	250	245	240	235	230

**Table 2:** DSC thermal analysis results for the studied composite materials.

Sample Code	Process I		Process II		Complex Melting Process			
	Vitreous Transition		Crystallization		Melting I		Melting II	
	T_onset_, °C	ΔCp, J/g·K	T_onset_, °C	T_max_, °C	T_onset_, °C	T_min_, °C	T_onset_, °C	T_min_, °C
0	1	2	3	4	5	6	7	8
M1	64.9	0.032	117.7	123.3	239.1	249	-	-
M2	64.7	0.076	117.1	124.5	237.8	249.9	-	-
M3	63.7	0.001	114.8	123.3	238.9	249.5	-	-
M4	64.2	0.051	108.3	117.3	236.1	248.9	-	-
M5	65.5	0.028	115.9	123.5	236.0	250.1	-	-
M6	72.4	0.044	116.6	124.1	153.3	162.9	237.9	247.4
M7	64.7	0.046	107.6	116.3	153.3	163.8	237.4	254.1
M8	74.5	0.010	114.9	126.1	153.7	163.6	238.5	252.0
M9	69.6	0.009	114.7	124.2	153.7	162.9	239.7	248.8
M10	73.7	0.001	111.9	123.4	151.4	163.2	236.8	247.1
M11	73.7	0.006	-	-	123.6	130.8	238.0	247.4
M12	74.0	0.045	-	-	123.6	130.4	238.6	247.0
M13	72.6	0.068	-	-	123.1	130.3	242.8	248.4
M14	72.2	0.006	-	-	122.7	129.3	240.1	246.9
M15	68.6	0.003	-	-	123.3	131.7	239.4	249.0

**Table 3:** The coding and composition of the obtained composite materials.

ME	Recycled PET (g)	Al Powder 800 nm (g)	Fe Powder 800 nm (g)	PP (g)	HDPE (g)	Total
M1	200	0	0	0	0	200
M2	190	10	0	0	0	200
M3	184	16	0	0	0	200
M4	190	0	10	0	0	200
M5	184	0	16	0	0	200
M6	140	0	0	60	0	200
M7	133	10	0	57	0	200
M8	129	16	0	55	0	200
M9	133	0	10	57	0	200
M10	129	0	16	55	0	200
M11	140	0	0	0	60	200
M12	133	10	0	0	57	200
M13	129	16	0	0	55	200
M14	133	0	10	0	57	200
M15	129	0	16	0	55	200
TOTAL RAW MATERIALS	2275.2	78	78	284.4	284.4	3000

**Table 4:** Determination of hydrostatic density.

Code	Average Mass (Mass)	Hydrostatic Density [g/cm^3^]
M1	1.755	1.318 ± 0.0004
M2	1.809	1.347 ± 0.0009
M3	1.923	1.382 ± 0.0011
M4	1.773	1.317 ± 0.0018
M5	1.845	1.381 ± 0.0004
M6	1.545	1.186 ± 0.0016
M7	1.546	1.395 ± 0.2833
M8	1.579	1.207 ± 0.0013
M9	1.701	1.306 ± 0.0000
M10	1.507	1.827 ± 0.6088
M11	1.517	1.180 ± 0.0004
M12	1.558	1.210 ± 0.0000
M13	1.577	1.219 ± 0.0004
M14	1.583	1.228 ± 0.0004
M15	1.840	1.318 ± 0.0004

**Table 5:** Water swelling test results for materials M1–M15.

Code	m_0_	m_1_	Q 168 h	m_2_	Q 408 h	m_3_	Q 504 h	m_4_	Q 600 h	m_5_	Q 1200 h
M1	0.0926	0.0928	0.22	0.093	0.32	0.0930	0.43	0.0931	0.58	0.0932	0.65
M2	0.0927	0.0930	0.32	0.0932	0.54	0.0934	0.76	0.0933	0.65	0.0933	0.68
M3	0.0928	0.0932	0.38	0.0933	0.57	0.0933	0.54	0.0935	0.75	0.0935	0.78
M4	0.0826	0.0828	0.24	0.0830	0.48	0.0831	0.61	0.0832	0.73	0.0832	0.73
M5	0.0970	0.0973	0.31	0.0975	0.52	0.0977	0.72	0.0977	0.72	0.0977	0.72
M6	0.0847	0.0849	0.24	0.0852	0.59	0.0856	1.06	0.0859	1.42	0.0860	1.53
M7	0.0772	0.0775	0.39	0.0780	1.04	0.0785	1.68	0.0788	2.07	0.0788	2.07
M8	0.0740	0.0746	0.74	0.0754	1.89	0.0755	2.03	0.0756	2.16	0.0757	2.30
M9	0.0774	0.0778	0.52	0.0785	1.42	0.0789	1.94	0.0790	2.10	0.0791	2.22
M10	0.0872	0.0878	0.69	0.0878	0.69	0.0882	1.15	0.0886	1.61	0.0887	1.72
M11	0.0796	0.0798	0.25	0.0803	0.88	0.0804	1.01	0.0804	1.01	0.0804	1.01
M12	0.0737	0.0740	0.41	0.0752	2.04	0.0759	2.99	0.0764	3.66	0.0765	3.80
M13	0.0806	0.0810	0.50	0.0812	0.74	0.0814	0.99	0.0815	1.12	0.0816	1.24
M14	0.0803	0.0832	3.61	0.0839	4.48	0.0840	4.61	0.0852	6.10	0.0852	6.10
M15	0.1038	0.1051	1.25	0.1051	1.25	0.1051	1.25	0.1051	1.25	0.1051	1.25
